# Origin of Insanity
*Etudes Médico-Psychologiques sur l'Aliénation Mentale. Par L. F. E. Renaudin. Paris: 1854.


**Published:** 1855-04-01

**Authors:** 


### Art. III.—ORIGIN OF INSANITY.*

The work of M. Renaudin must not be regarded as a complete systematic treatise upon Mental Alienation. It is true that we possess several works which embrace more or less fully all the subjects usually considered in general descriptions of insanity, and which do not equal in bulk the book now before us. M. Renaudin has devoted this work to the elucidation of some particular questions only, of the

* Etudes Médico-Psychologiques sur l'Aliénation Mentale. Par L. F. E. Renaudin. Paris: 1854.

highest importance indeed, and indissolubly connected with the very foundation of psychological medicine. Were we to attempt to give the general character of this work, we should describe it as a laborious philosophical disquisition upon the etiology of mental diseases. No part of the great subject of insanity can be more important than this, none more urgently needs elucidation, and none more demands the highest powers of the psychologist and the practised skill of the physician. In the execution of our task of presenting our readers with an account of M. Renaudin's labours, we shall certainly fail in conveying an adequate conception of the great acumen, accurate reasoning, and extensive research displayed by the author. But we may hope to succeed in compressing within the narrow limits of a review his leading ideas, and to exhibit some of their most important bearings. We also believe that those who shall derive an imperfect apprehension of the interest of M. Renaudin's essays from our analysis and observations, will seek in the original for fuller satisfaction. In this event, our failing will be as serviceable as our success.

The fundamental principle of M. Renaudin consists in the proposition that it is impossible in the study of mental alienation to disregard either the psychical or the somatic element of man, and that it is equally irrational to attempt to exaggerate the importance of either element at the expense of the other. It is impossible, he observes, to isolate the moral being from the psychical being; man is a psychico-somatic duality which must be considered in its entirety under the penalty of falling into the most serious errors. Every other manner of seeing only ends in the creation of a phantasy which bends more or less to such and such a system, but which never represents the entity, from which it always differs in some salient point. When man is insane, it is not by a single part that he goes wrong; all his existence is more or less interested. He is *diseased* in the physical as well as in the moral element, in different proportions in different cases, but never to the exclusion of either of the two elements which make up one existence. It is on this fundamental idea that depends the differential diagnosis of insanity; and it is by taking this for our point of departure that we shall succeed in disengaging from the midst of the odd existences of which society presents such numerous examples, the pathological element of ordinary eccentricities or of criminal perverseness. It is on this principle of psychico-somatic duality that rests the appreciation of the many phenomena that present themselves to us in a lunatic asylum. In all the phenomena of normal psychical life we observe two features so inherent to the human nature that every language possesses special terms for the irdesignation. We mean *sensation*, and the transformation of sensation into conception; *re-action* and its transformation into action. It is in the mode of evolution and of succession of these facts that we discover certain pathognomonic signs, by the aid of which diagnosis may acquire a degree of precision especially indispensable in medico-legal inquiries. In the state of reason there is a perfect correlation between the four terms of this proportion, which is the general formula of our existence. The error that may arise under the influence of one of them is corrected by another, when the physiological state is normal. From this basis is deduced the *definition* of mental alienation, which M. Renaudin takes to consist in a lesion of the sensibility or in its abnormal exercise, over-ruling more or less the patient's will, and destroying wholly or in part the moral freedom, the basis of reason and of all moral responsibility.

The author thus expresses the differential diagnosis between mental soundness and unsoundness:—

"An opinion is stated; reason demonstrates that it is the expression of truth; it finds, however, persons who will contradict it. Tax them with ignorance, bad faith, you may; but it cannot be said that they are madmen. Their arguments are absurd, paradoxical; they enjoy their moral liberty. If they re-act but imperfectly upon their impressions, they still re-act; if they give themselves up to their errors, it is in the full exercise of their will. When, in consequence of a particular state of the digestive organs, the organ of taste does not perceive flavours in the ordinary manner, but that in announcing this abnormal sensation the patient recognises its dependence upon the organ, and not upon a change in the nature of the object, there is only partial lesion of the sensibility; there is not even error, and still less mental alienation. But if the patient displaces the error, if the psychical element does not correct it, and he admits that this flavour belongs to the object itself; if, believing in poisoning, he so far loses his moral liberty as to wish to be revenged upon the supposed authors of this imaginary crime; if, under the influence of his ideas, his affective sentiments are disturbed, the lesion of the sensibility is then complete, and insanity is easily recognised. It is true, then, from this example, that a man is rather insane through defect of re-action against error than through the error itself, for the patient no longer reflects the impression, he bears it without control, and abandons himself to it without reservation."

Such is an instance in which the origin of the error lies in the somatic element. Take an example in which the psychical element is the point of departure:—

"A man is odd, eccentric, pusillanimous, or proud, without judgment enough to give the right signification to the facts passing around him, nor enough discretion to regulate his instincts and inclinations. Under the influence of this idiosyncrasy there arises a passion which establishes for a moment a state of equilibrium; it is upon this passion that depends the direction given to the sentiments and to the affections, which are as variable as the stimulant. His entire existence is organized upon this incongruous assemblage, without our being able to say that there is insanity in the nosological meaning. Such a man is properly held responsible for his acts, so long as no pathological lesion complicates his situation. But if these irregularities of conduct go to the extent of modifying his constitution, if hallucinations become the point of departure of his determinations, his originality becomes exaggerated and pathological, because his sensibility is involved in its double psychico-somatic relation. These facts show that insanity may spring from the irritation of each of the two elements, and that one or the other may dominate in turn, according as it is either cause or condition of causality. But mental alienation is confirmed only when lesion of the general sensibility is present."

Proceeding from this basis, the author goes on to trace with great minuteness the development of psychical life, the conditions of causality, the transition from reason to insanity, ideas and their relations with the general sensibility, instincts, wants, the development and formation of ideas, and the types of mental alienation. These subjects of course involve the elemental questions of psychology, and we may spare a detailed exposition of the author's views upon these by observing that they agree closely with those of Reid. He admits fundamental ideas,—that is, ideas which stand upon the foundation of common sense, and secondary ideas deduced from the first by judgment and discernment.

Concerning the types of mental alienation, he observes :—

"If the insane present general characters by which the perturbation which governs them is recognised, observation teaches us that the intellectual disorder is not the only pathognomonic symptom, and that insanity constitutes a group of fundamental types having special characters and course. However varied the phenomena that accompany or constitute the delirium of the madman, certain primordial aberrations are detected, around which turn the secondary symptoms peculiar to the individual. It is in the reciprocal relations of the sensibility and the reaction, that we find the elements of the nosological table of mental alienation."

Following Esquirol and other authors of the French school, he recognises four fundamental types : monomania, lypemania, mania, and dementia. Monomania is chiefly marked by a surexcitation of the sensibility, producing an energetic re-action, which exaggerates spontaneity, the feeling of personality, and modifies the psychico-somatic relations of the subject with the outer world. It is under the influence of this state that certain expansive passions, reaching a pathological development, assume the direction of delirium, involving errors of personality which, binding the moral freedom, become the exclusive motors of the will. This delirium is more or less organized according to the intellectual aptitude, and in some subjects it puts on all the characters of a mortifying logic. If in this form of mental alienation we almost always observe the same organic lesions, we must nevertheless admit that the psychical element predominates, and that in a somatic point of view the delirium may be considered as more exclusively cerebral.

In lypemania there is also surexcitation of the sensibility, but this is painful, and often proceeds to stupidity, a sort of chronic spasm which suspends for a greater or less length of time the most important functions of the psychico-somatic existence; re-action is incomplete, insufficient, or wanting.

If we examine the general phenomena of insanity, we are at first tempted to admit but two principal forms, which seem to include all: mania and dementia. But more careful analysis reveals that mania bears a peculiar stamp which constitutes it a distinct type. The most frequent form of mental alienation, mania, is especially characterized by disorder of the sensibility, to which corresponds an analogous disorder of the re-action, in which the mobility is principally noticed. It is the anarchy of the passions and sensorial functions.

In dementia the spectacle is very different. The sensibility is either blunted or restricted, the re-action is almost null. Physical lesions predominate, and animality has assumed the upper hand. Intellectual life has disappeared under the influence of a vegetative existence.

M. Renaudin having thus laid the basis of his psychological system, both physiological and pathological, proceeds to investigate minutely the etiology of insanity. And upon this point we may take the opportunity of observing, that it is in the profound study of the remote, as contradistinguished from the immediate, causes of insanity, that we must look for the most accurate and scientific extension of our knowledge of mental pathology. It must be obvious to all those who have devoted their minds to this subject, that the path hitherto pursued in the accumulation of what are presumed to be etiological data is one pregnant with error. In the etiological tables systematically kept in most of the continental and British asylums, the proximate exciting cause is chiefly kept in view. Poverty, reverses, mental concussions, physical lesions, and other circumstances apparently connected closely in point of time with the outbreak of open insanity, are set down as the causative agencies. An imposing statistical battery is then formed by the enlistment of the figures contributed by a number of different asylums into one aggregate. Any doubts that might be entertained as to the value of similar data in the elaborating of etiological laws, when drawn from a single asylum, and confined to a limited number of cases, become surprisingly dissipated when we are confronted with the formidable array of figures drawn from many asylums and some thousands of cases, all made to swell the different heads. There is nothing so hard to contend against as so-called statistical evidence. He who resolutely resists the conclusions put forth by many ardent advocates of the numerical method—conclusions which it is triumphantly announced rest upon facts multiplied to the extent of eliminating the possibility of error—is apt to be decried as one impenetrable to the plainest of all demonstration. We will not for a moment call in question the immense value of the numerical method in the determination of all questions to which that method can be rigorously applied, under all the necessary logical conditions. The first and most absolutely required of these conditions is, the accuracy of the individual facts registered. What if there be any fallacy here at the starting point? Can any accumulation of individual errors make up an aggregate of truth? And to secure precision in the primary facts is a difficulty hard to overcome. We have no concern in this place with the application of the numerical method to problems in general pathology. We believe it possible in many questions in general medicine, such as the mortality from particular diseases, and possibly the value of certain modes of treatment, to register and collect a sufficient number of homogeneous facts to warrant definite conclusions. But we very greatly question whether it be, at the present epoch of our knowledge, possible to apply such a system to the investigation of psychological questions without imminent danger of arriving at false conclusions, and by so doing, of erecting a series of arbitrary conventional dogmas, which, so far from facilitating the attainment of truth, would have the opposite effect of concealing it from our sight, by filling the mind with a specious but false idolatry. Let us take a familiar illustration. In etiological tables we sometimes find intestinal worms assigned as the causes of certain cases of insanity. It may be conjectured that in some of the cases so classified, insanity would not have declared itself had not the intestinal worms been present; so far, intestinal worms may with propriety be said to have been the cause of insanity. But if we analyse a little further, and reflect that many persons have intestinal worms for a very lengthened period without exhibiting any disposition to insanity, we are driven to the conclusion that there must be some antecedent condition in the subject, some peculiar modification—to resort for the occasion to M. Renaudin's phraseology—of the psychico-somatic constitution, predisposing him to become insane, if brought within the operation of sufficient determining agencies. We must believe that in the case of a person having such a proclivity, the influence of intestinal worms is purely accidental, and that if he be supposed to escape this particular influence, then he might fall under the influence of some other conventional cause—as grief, for example—and then, under the actual arbitrary etiological tables, the same radical psycopathy would have to be transposed into another column. And thus *decipimur specie recti.* It is in a similar manner asserted that intestinal worms cause epilepsy. But how many persons are there who, being afflicted with worms, yet never become epileptic? There is here also an antecedent idiosyncrasy, an epileptic actuality, without which neither worms nor any other circumstance will give rise to epileptic fits.

It may, indeed, be urged that, although the registration of circumstances associated in time with the outbreak of insanity may not lead to any accurate understanding of the essential conditions of insanity, it may have its use in indicating those obvious disturbing agencies which favour the development of insanity. Such being well determined from tables of this kind, the physician has acquired valuable indications in practice. We doubt whether, even with this limitation, it is not giving undue prominence to the class of accidental agencies, or causes, to make them the heads of etiological columns. The physician knows well enough, without the aid of statistical tables, that intestinal worms may lead to the development of various pathological conditions. Whenever such a case comes before him, whether he apprehend epilepsy, insanity, or any other disease, he will expel the worms. The study of secondary causes by means of statistical tables, therefore, does not lead much nearer to the real object the psychological physician must ever have in view, namely, the discovery of the latent pathological substratum, the insane actuality existing, perhaps, from birth, and often transmitted from generation to generation. The influence of heredity has not, indeed, been overlooked; but we doubt whether it has been rightly appreciated, and we do not doubt that much, very much, still remains to be done in detecting the genesis of mental aberration in persons who may be supposed to be free from hereditary stamp. The primary influences which create the insane actuality are often lost, obscured by lapse of time, unobserved, not remembered, and the mind of the physician, hankering as the human mind will for the definite, fixes upon a later period in the development of alienation, and seldom fails to associate some marked mental aberration and tangible exciting circumstance; and to behold in them a relation of cause and effect. But how much of insanity might there not be, and how many mind-impairing circumstances in operation, *prior* to the date at which he took up the psychological history?

It is in the minute and philosophical analysis of the insane constitution, and of all the elemental causes which assist in the genesis of insanity, that lies the great merit of M. Renaudin's work. He begins by discussing the subject of hereditary predisposition; he considers the influence of temperament, character, the intellectual aptitude; the influence of age; the predispositions proper to each sex; and the influence of education on the physical and moral development. We will cite a passage on education which is especially interesting.

"Man is not a *tabula rasa* upon which we can inscribe any characters we please. Anteriorly to all education, there exists psychico-somatic predispositions which must be taken into account; and many friends have had to pay dearly for the adoption of an *à priori* exclusive system settled before the birth of the child. From the commencement education is exclusively physical, and is summed up in two primordial indications, which must never be lost sight of, even throughout the whole course of existence; the normal and harmonious development of all the parts of the organism, and the direction of this development into habits fitted to ensure its regular play. We must at first avoid opposing to the aimless mobility which characterizes the infant a coercion antipathic to its nature, and more calculated to increase its native irritability at the expense of the locomotive system, the development of which ought to be encouraged in order to counterbalance the predominance of the nervous system, which is too easily impressionable at this epoch of life. It is to the defect of equilibrium between those two essential elements that we must attribute those numerous nervous and convulsive affections of early infancy which, as much as heredity, are a predisposition remote, but almost certain, to the ulterior development of mental alienation."

The influence of education is admirably discussed. If we were, however, to hazard an opinion upon the author's doctrines upon this subject, we should be disposed to object that he has exaggerated the effect of education in the production of insanity. That a badly-directed education may tend to develop unduly either the mental or physical element at the expense of the other cannot be doubted, but that mental alienation should frequently take its origin from this point of primitive departure is not easy of demonstration. Even in cases supposed to be of this nature, were a rigorous analysis possible, it is highly probable that we should, for the most part, discover an antecedent insane actuality, rendering the individual peculiarly susceptible to the operation of disturbing causes. To maintain that education has an absolute potential influence in the production of insanity is to maintain that all mankind are hanging in the balance between sanity and insanity. It is certain that no amount of error and vice in education will evoke positive insanity in some organizations.

In his exposition of the intellectual and affective elements of the human mind, M. Renaudin wisely abandons the attempt to adhere to a refined classification and isolation of the different passions and modes of thought. "Man," he says, "is one indivisible whole, and whatever care we may bring to the analysis of his sentiments and reactions, we only succeed in isolating them for a moment, in order the better to recognise the intimate union and reciprocal dependence of the two elements of which he is made up." He, therefore, confines himself to the study of the succession of phenomena rather than to their classification. An able chapter is devoted to the faculty of reason, free-will, and the question of responsibility. He thus explains the reasoning power so often observed in the insane.

"It is an incontestible fact that the monomaniac or the lypemaniac reasons and follows, in the thread of his delirious ideas, the path of ordinary reasoning. Far from undergoing, in this pathological condition, any impairment, the intellectual aptitude seems, on the contrary, to concentrate itself with so much energy that, during the period of incubation, the patient makes of his existence two distinct parts. The one has all the appearances of reason; its relations with the external world are preserved with extreme care. In the other, on the contrary, which remains impenetrable to all, the delirium becomes completely organized, in order to declare itself at a moment when it is no longer time to arrest its course."

How many illustrations do we not see of this condition! There are, even beyond the walls of lunatic asylums, not a few men who exhibit to the world one phase only of their mental existence, and who cherish for themselves alone an ideal life full of extravagance, of absurdity, of actual insanity.

In another chapter the author examines the influence upon man of his relations with the surrounding world. Under this head he surveys the bearings of civilization, celibacy or marriage, political institutions, arts, trades, the hygiene of various professions, changes of fortune, and religion. The limitation he assigns to these agencies is one which we could have wished him to have assigned to education. He says, without regarding the surrounding medium as a direct pathogenic agent, it must be considered to be a predisposing condition to some virtualities. Whilst admitting this influence, however, he says, it is subordinate to the reaction of the subject, and however bad the conditions of the medium may be, it is rare not to find in him a compensative prophylaxis against his dangers.

In the sixth chapter the author traces the psychical signification of the different functions as well as of their principal modifications. After some general reflections upon the different functions, he examines the influence upon the mind of alternate activity and rest; the relations of the nervous functions of the circulation; the psychical signification of the organs of sense; that of the digestive functions; of the abuse of alcoholic drinks; of the generative functions. It is so true that the genesis of insanity must be sought for in physiology itself, and its development traced, step by step, through the apparently most trivial deviations from health, that we may frequently discover in the normal varieties of functions the starting-point of hallucinations and of delirious conceptions. This subject, so important to the full understanding of the etiology of insanity, and essential to a right interpretation of its phenomena, is treated by the author with singular accuracy of description and great acumen in tracing the bearings of functional irregularities upon mental health and disease. With the same power and success he advances nearer to the consideration of the immediate sources of insanity in his appreciation of the psychical signification of the pathological modifications of the different functions. Indeed, it is in the reaction of various morbid states of the different functions upon the mind that we frequently witness the transition into insanity. In discussing this subject the author is naturally led to estimate the spiritual and somatic doctrines of insanity. The exposition we have already given of his leading principles furnishes the clue to his views upon this question. He is neither exclusively spiritualist or material. The two elements are affected together, and the one cannot be isolated from the other. He refers to the principles of moral treatment of Leuret, which, he says, have had the effect of postponing the investigations of a wholesome nosology. The natural reaction against this conclusive doctrine led to the opposite extreme, so that many physicians, adopting a materialism no less exclusive, believed that they should find in pathological anatomy the secret of the disorder presented by the delirium. This was to fall into error, for it was running the risk of confounding effect with cause, and of connecting the intellectual disorders with certain lesions which had only become developed under the influence of insanity. Where a moral impression is experienced, it has, in the first place, a physiological reaction upon some part of the economy, then a pathological reaction, and upon this depends the invasion of insanity. On the other hand, all pain, every pathological modification of the functions, has a psychical correlation which is only fatal when the moral force yields. It is, therefore, in this pathological condition, in some sort mixed, that lies the knot of the question. According to the definition of mental alienation given by the author, we have seen that the primordial phenomenon consists in a modification of the general sensibility. He therefore applies himself to the task of studying how disease in general can modify sensibility, and what is, according to the nature of certain special diseases, the mode of perturbation, direct or sympathetic, which may result. We must refer the reader to the work itself for the distinction between the delirium of fever and the delirium of the madman. The hallucinations which appear in the delirium of fever have a peculiar character, which does not permit them to be confounded with those of the really insane. This character is more especially somatic.

The subject of hallucination is investigated at great length. He points out that illusions and hallucinations have often been confounded, and thus sets forth the diagnosis. Illusions and hallucinations have this in common, that they constitute erroneous perceptions; the sensation is real, the perception is inaccurate; and when there is alienation the perception is still more inaccurate. There is illusion when the individual assigns to external objects characters and forms which they do not possess. It is an error of objective perception. In hallucination, properly so called, the object or external agent does not exist, and the sensation is perceived just as if they were in relation with the economy. It is an error of subjective perception, often confounded with instinctive predominance. In order that illusion may exist, it is necessary that there be external to us a real object that strikes the senses, and that in consequence of an objective sensation, more or less complete, there be erroneous perception. This error of perception has its origin in our senses, in the object itself, or in the medium which separates it from us. Examples of illusions depending upon the last cause are of constant experience. Hallucination, on the other hand, a perception exclusively subjective, has its origin in the reciprocal reaction of the sentient extremities and of the centre of perception. It recognises for cause or condition of causality the physical influence, as well as that of the psychical element. The subject of hallucination *feels*, but the cause of his sensation is in himself, instead of being beyond himself; no object strikes his senses, and he is persuaded that everything he experiences results from the impression produced by external agents. Two fundamental facts must be carefully distinguished in this case: there is either a modification of the sensibility by excess or defect, or else there is error as to the cause of the sensation. One takes a rheumatic pain for the result of violence of which he is the victim. Reduced to these terms hallucination might be confounded with illusion; but these misunderstood victims see the individuals who torment them, hear their voices, and even in secret are present at the conspiracies laid against themselves. Are you deceived as to the nature of a sound heard, that is an illusion; do you hear a sound that does not exist, that is an hallucination. If a person speeds along the streets, thinking he is pursued by all those who are walking behind him, if he supposes their pace more hurried than is really the case, he is the victim of an illusion: but if, under the influence of a delirious preoccupation, he imagines himself to be insulted by persons who are silent, and hears distinctly expressions that no one utters, there is hallucination. In the first case there is only an erroneous interpretation; in the second, there exists, beyond the individual, nothing to interpret, everything passes within himself.

But "hallucination, or rather the hallucinatory condition, does not constitute mental alienation, nor can it form a distinct species in this nosological family. Preceding or following the invasion of the insane, delirium it is not a constant essential symptom; and if we observe it in the outset, or in the course of the affection, we may ascertain that it is far from possessing that character of persistence which is the mark of every typical affection. Limited, in many instances, to a simple diseased virtuality, it often presents nothing by which it can be distinguished from what is observed apart from all delirium. It is, therefore, when it exists an element of delirium without being the delirium itself, and if it be sometimes an essential condition of causality of this, that depends either on the surrounding medium, or on special circumstances connected with the idiosyncracy of the subject. It is, if I may so express myself, central or peripheral; and we find its primordial evolution either in the psychical or in the somatic element. Whichever be the point of departure, it only becomes a fact in mental pathology when there is simultaneous or successive action of both."

In this country, and especially in our courts of law, whenever the question of insanity is raised, there is nothing so much insisted upon as the existence of something which is referred to as "delusion." Many lawyers take the presence or absence of delusion as their decisive test of mental unsoundness or soundness. But no definition they have furnished of the term is capable of practical application. They call for evidence of that which they cannot define. In most *civil* cases they would not hesitate in pronouncing that person insane who should exhibit marked morbid sensorial hallucinations; in *criminal* cases, the same conclusion would be less readily admitted. Of all the various mental phenomena which are confounded amongst us under the common name of delusion, none is considered more undoubted proof of "delusion" than hallucination. But medical psychologists, who carry the analysis of mental operations further than lawyers, have well ascertained that something more than delusion, as the term is commonly understood, is necessary to constitute insanity. There is something beyond; it is that something which it requires the skill of the medical psychologist to discover and to decide upon. Leuret has well expressed the difficulty in the following words:—

"I have not been able, whatever pains I have taken, to distinguish by its nature alone a sane idea from a reasonable one. I have sought at Charenton, at Bicêtre, at the Salpêtrière, for the idea which seemed to me the most mad; then I have compared it with many of those which are current in the world, and I have been astonished and almost ashamed at not perceiving any difference."

The same difficulty is also admirably set forth by our author. If it were possible to regard mental alienation solely from a metaphysical point of view, it would then be possible for lawyers, as well as physicians, to enter upon the task of diagnosing unsoundness of mind, with an equal prospect of success. But that is not possible; and since in every case of insanity, the physical as well as the psychical element is compromised, and that the psychical phenomena are always directly modified by the changes in the physical structure, one cannot avoid a feeling of astonishment at seeing men necessarily ignorant of the structure of the human frame and of its relations to the mind, pronouncing authoritatively the most absolute dogmas upon the most difficult points of general mental pathology, and delivering the most unhesitating decisions upon the sanity or insanity of individuals whom perhaps they have scarcely seen.

Proceeding with his subject, M. Renaudin traces the *mode of manifestation* of insanity. He denies at the very outset the very common opinion that all insanity consists in frantic raving and tumultuous agitation. Fury, he contends, is only an accident, a complication, and by no means the essential character even of mania. He quotes, with approbation, a passage from Esquirol, which, as it conveys a great truth, is reproduced:—

"It is through having mistaken fury for insanity itself, through having attributed to this symptom a great therapeutical importance, that so many grave errors have been committed in the treatment of the insane. The furious were bled to excess, with the intention of reducing their strength, and it was not perceived that the loss of blood increased the evil, that it only calmed the patients by depriving them of the power of reaction necessary for the resolution of the disease. * * * Since these unfortunates have been treated with benevolence, the number of the furious has diminished to that extent, in asylums properly conducted, that out of several hundred lunatics sometimes not one is seen in a state of fury."

M. Renaudin adds, that fury had vanished from the asylum of Maréville, which he governs, since they have ceased to take it into account in the internal organization of the establishment.

He observes that the course of mental alienation offers difficulties which it is important to signalize after having disengaged them from the complications which to some extent masked the fundamental phenomena. In the most regular succession of its symptoms, insanity presents itself at first in an acute condition, and the observer detects a reunion of phenomena, having a relation to the type it has definitively assumed. Then follows a transition-period, which terminates either in recovery, in dementia, or in a chronic condition. This chronic condition, which is, so to speak, a quite peculiar mode of existence, may indeed sometimes resolve itself by a crisis, but it usually persists so long as no morbid modification has been wrought in the constitution. If in its essence mental alienation is a continuous affection, we still remark in the manifestation of its symptoms a kind of periodicity, which is often a precious indication in therapeutics. Remission, he says, is a frequent phenomenon; but he strenuously and at some length argues against the existence of *lucid intervals*. He observes, that in every disease, acute or chronic, as well as in the physiological condition, permanency of the manifestations is a very rare exception, and we are not to be astonished if for lucid intervals we are called upon to substitute remissions.

The author then passes on to the symptomatology of insanity. He discusses the usually received fundamental forms of insanity which are made to serve as the basis of systems of classification. Upon the subject of monomania he puts forth views not yet generally admitted, but which are every day receiving the support of new adherents. He contends that there is no such thing as a monomaniacal entity of which any particular act is the only pathognomonic sign. He refers to an essay published by him in the "Annales Médico-Psychologiques" in 1844, in which he demonstrated that homicide belonged to all the types of mental alienation, and that the homicidal monomania was an illusory entity, without foundation and without identity of character, when the act is considered as the only and fundamental symptom. Still, accepting the term monomania, with some modifications, he expresses his adherence to the classification of Esquirol.

Speaking of the *terminations* of insanity, he dwells with some minuteness upon the doctrine of crises. A crisis, he says, is either moral or physical, and often is of a mixed character. Again he follows Esquirol in contending that recovery is possible only by means of a crisis, not only pathological, but equally psycho-physiological; and that it is marked by a more or less gradual return of the normal physical and moral functions. We have not space to follow our author throughout his able exposition of the different critical phenomena. It is, however, one of the most interesting features of his work, inasmuch as it is from a close and correct observation of the physical and psychical critical phenomena that he seeks to derive his most valuable therapeutical indications.

The concluding chapter, one of great length, is devoted to the analysis of the four different types of mental alienation. These four types, the same that are recognised by Esquirol, constitute truly distinct pathological entities. The elements of a differential diagnosis abound. The author here unfolds his reasons for limiting the application of the term monomania. The monomania of theft, of homicide, of suicide, of arson, erected into distinct entities, without precise pathological characters, and rejected by legal tribunals, have raised against true monomania, a reaction against which it is the author's study to contend. He refers to the writings of Foville and Falret, who appear almost to discard monomania altogether. "I. Frank, again, rejects monomania, and admits only two types, mania and dementia. He is thus driven to the subdivision of mania, and is obliged to admit that in chimerical mania the patients are so carried away by their chimera, that they regulate all their actions according to the part it enacts. In every other respect their reason is scarcely impaired; even more, in all that does not relate to their fixed idea, all the faculties of the soul are often healthy, and sometimes even excellent in part." M. Morel, attaching a very restricted meaning to the word monomania, and resting on the incomplete definition often given of this type, lays down as a principle the *physiological impossibility of an alienation bearing upon a determinate idea with an intact conservation of the reason of the individual on all other points.* He therefore, like I. Frank, rejects monomania as a type, and confounds it with mania, with which it becomes a variety under the name of *systematised mania.* But, although not sharing the ideas of M. Morel as to typical classification, he admits that the solidarity of the psychological functions is for him a fundamental principle, but contends that it is compatible with the admission of monomania as a type. M. Delasiauve maintains that the terms monomania and lypemania no longer satisfy the necessities of science, and convey no precise meaning. He comprises under the name of partial delirium a great order, having its varieties, amongst which, occupying the first place, is a true monomaniacal delirium, consisting in *the alteration of a sentiment or the predominance of a false conviction, compatible in every other point with the free exercise of the faculties.* M. Renaudin objects to this view, that besides the predominance of a particular sentiment, we must take into account the reaction of the sensibility, and not forget that activity or depression impart to this sentiment very different directions. It is for this reason that he is inclined to maintain the opinion which distinguishes monomania as much from mania as from lypemania. The author expresses himself of the same opinion as M. Baillarger. He praises the precision with which the physician of the Salpêtrière sets forth his opinion of the essentiality of monomania, which consists not only in the isolation of a delirious conception, but in the concentration of a series of dominant ideas, and in not changing its form, whatever the accessory phenomena and the number and variety of the secondary false ideas. Its evolution comprises delirious conceptions, hallucinations, unwonted or uncontrollable impulses, either isolated or combined. Monomaniacs live in their delirium under the mask of the healthy mind; and monomania must so much the rather be admitted, because it is the fundamental type of insanity, and is connected less than the other forms with so-called organic lesions. To this M. Renaudin adds, that if the psychical element seems to predominate in monomania, it still only exists through the complementary influence of the somatic element; and if primitive organic alterations are rare, the functional dynamism is a powerful condition of causality upon which the varieties of evolution are based, from the instinctive impulse of material want, up to the ecstasy which is completely freed from all contest with this want.

The author then enters upon the discussion of the general and differential characters of monomania, chiefly resting upon the analogies existing between the state of reason and the pathological state. He cites several interesting cases to show that the first origin of monomania is sometimes detected in the somatic element, which may lead to the organization of a monomaniacal delirium prepared apart from the sentiment, but fully confirmed from the moment that this shares in the disorder. As to the ideas, he admits with Leuret that they are far from possessing in themselves a pathognomic character. Neither the idea nor the act, which are its sensible signs, constitute the disease which is in the domain of the sentiment. It is only upon this condition that we can diagnosticate monomania, not in its final result, but in its pathogenic mode. As to the diagnosis from lypemania, it is impossible to confound the sometimes ingenious comparisons of the lypemaniac, with the active reason, almost rising to inspiration, of the monomaniac. The lypemaniac shuns, eludes, avoids; the monomaniac goes forwards and becomes a creator. From whatever side, then, we regard the question of diagnosis, we find that it is resumed in the study of the evolution of the sentiment, and we discover the evident proof of the essentiality of a type which we have proposed to call *hyperphreny*, and for which we may without inconvenience preserve the name of monomania.

In studying the etiology of monomania, M. Renaudin observes that the evolution of monomania depends more upon groups of causes than upon special causes; and that in this type, as well as in the others, we must for the most part attach more importance to the conditions of causality than to the accidental circumstances which have coincided with the invasion or manifestation of the disease. If the hereditary transmission of mental alienation be a well-determined fact, the transmission of the special form is far from being well established; and the author has known many families, the members of which presented every variety of the nosological scale. The indirect hereditary predisposition ought to be taken into more serious consideration. We often meet in the world with individuals who make themselves remarkable by their originality, their oddity, doing everything in opposition to other men, and having a peculiar manner of feeling and of expressing their sensations. The predominance of the personality regulates in them all the sentiments, and everything leads us to conclude that should insanity declare itself, it will take the form of monomania. It is in this that indirect hereditary predisposition consists, and is principally revealed by peculiarities of constitution, of character, of inclinations, the transmission of which cannot at the present time be doubted. But these elementary conditions of causality would often be without influence, if different circumstances did not arise to give them strength.

The nosology of monomania is then considered. It is not sufficient to diagnosticate a monomania to be satisfied that we have said all. Side by side with the general features, we have the individual features, and if each one is monomaniac after his own manner, we, nevertheless, observe different tendencies common to some, wanting in others, although all present the general impression of monomania. Hence the necessity of establishing certain important distinctions, not only in a nosological point of view, but also as regards legal medicine and therapeutics. The author then proceeds to relate a number of interesting cases, as illustrations of the different varieties of monomania. The first form described is ambitious monomania. He observes that the abuse of alcoholic liquors and good living is perhaps the most dangerous cause; in addition to the permanent surexcitation of the brain, this abuse entails a variety of affections in the different organs, and those of the heart are not the least frequent. It is especially this disorder of the circulation which has appeared to some physicians an indication for bleeding, a fatal practice, to which many cases of general paralysis must be attributed. Amongst the patients the author has seen, several have reached through this course to the belief that they were the Supreme Being. All had become incurable, because, in giving a too exclusive attention to the disorders of the circulation, alcoholic intoxication had not been sufficiently considered. Ambitious monomania has also sometimes broken out under the impression of direct physical causes, when it has not been possible to connect this form with any anterior disposition depending on the character or education.

Ambitious monomania, according to M. Renaudin, is daily becoming more and more rare, although the number of the insane is far from having fallen off.

Religious monomania is the next in order. It is the prevailing sentiment which determines the character of the monomania, since it becomes the regulator and the motive power of this new existence. This existence moves henceforth in a circle of errors by the same processes which formerly placed it in relation with truth; and it is in order well to appreciate the different phases of this pathological condition, that we must distinguish in it three *moments*, which have a real importance, both in a medico-legal and a therapeutical relation. The sentiment becomes isolated in abstraction; it enslaves all the other sentiments, or confounds itself with them by yielding especially to the sentiment of personality; and lastly, in the third *moment*, the access of the prevailing sentiment is revealed by a negation of the others, that is, besides the transformations of the personality, we find also perversion of the affective element, or of some other. This truth, partially seen in studying ambitious monomania, is still more evident when we enter the sphere of religion. Here, as in the preceding form, it is less by the fundamental idea than by its applications, its evolution, and its sentimentality, that the delirious virtuality can be appreciated; for what is the idea enunciated by the religious monomaniac, of which the element cannot be discovered in the sacred writings? M. Renaudin relates in this chapter several remarkable examples of this form of mental alienation, in some of which, as in the case of Swedenborg, the hallucinations and delirious conceptions of the patient were received as sacred truths by persons not insane.

*Affective monomania* is then considered at some length, and its diagnosis determined according to the general principles laid down by the author.

The concluding part of this excellent work is occupied with an examination of some of the important medico-legal questions at issue between the professions of law and medicine. The author refutes with admirable force the doctrine of M. Molinier, the professor of law at Toulouse, who dwells upon the necessity of condemning the monomaniac, on the ground that society possesses no preventive measures with respect to him, and that it would be utterly without defence if it did not oppose to him repressive measures and the fear of punishment. According to M. Molinier, this fear is so efficacious and so salutary, that he attributes to it the triumph which some patients have obtained over their impulses, a triumph, which, M. Renaudin observes, can only be explained by the integrity of the will. The argument of M. Molinier is fundamentally the same as that often enunciated in our own courts of law; and is equally based upon an imperfect knowledge of the varieties and essential characters of mental alienation. M. Renaudin's arguments in favour of medical *versus* legal interpretations and judgments in matters pertaining to mental diseases, may be summed up as follows :—

1. Mental alienation cannot be considered as being ever *partial.* It either is or is not: its type may vary as happens in ordinary diseases; and the more or less extensive sympathetic complications resulting from it depend upon the initial pathological conditions upon the appreciation of which rests the diagnosis of the affection.

2. Monomania is a delirium having characters peculiar to itself. It is built up from a pathological condition which, if it borrow much from anterior predispositions, constitutes from the time when it is organized a morbid idiosyncrasy subject to special laws, at the same time as to the ordinary physiological laws.

3. The idea does not constitute the delirium,—it is the expression of it,—and represents the delirious conceptions, which must be judged not only in their relations with the primitive virtualities, but especially with the pathological element with which they are associated.

4. It is in this only that resides the *criterium* of their influence upon the determinations which reveal themselves, either by restriction or extension. The delirious conceptions may by a certain sentimental elaboration aggravate the initial pathological state, but it is commonly in the exacerbations of the latter that they produce their extreme consequences, and during its remissions sometimes only the obscure virtuality remains. It is, therefore, not by the characters of the period of remission that monomaniacs can be judged.

5. Monomania being not a passion, but a well-established pathological condition, the acts committed under its influence cannot constitute a punishable infraction, whatever appearances of discernment may exist. The loss of the will may perfectly well be conciliated with the consciousness of this loss; and it must not be lost sight of that the bases upon which the apparent discernment of the monomaniac is exercised, differ from those upon which common reason reposes.

6. The tribunals must therefore seek in the reports of physicians not only for information as to physiological and pathological facts, but also for the legal appreciation of the psychical value; and from the moment that mental alienation is established, whether it assume the monomaniacal type or any other, moral irresponsibility is the necessary consequence.

We have now glanced at most of the leading topics of M. Renaudin's first series of *Études*, preserving very closely the order in which they have been taken up and developed by the author. Many interesting facts and arguments, having a more or less immediate bearing upon the subject actually under consideration, are dispersed throughout the book. Most of these we have necessarily passed over. We are tempted, however, to bring together some observations upon the effect of isolation on the mind, and on the influence of the cellular system in the treatment of insanity, on account of the great practical importance of the question in the construction and regulation of asylums and prisons.

Every individual who isolates himself is very near the confines of insanity, either by going beyond the ideas of the times, when he loses himself in the uncertain, or by exaggerating his individualism, when he opposes his isolated strength in open conflict with the collective force. Beneath the weight of these influences many succumb. The moral sense must be wonderfully developed that can elude this catastrophe; and we well know that it is the exception. It is in the isolation of this force that lies the first step to mental alienation. . . . . Many insane patients are incoherent only when left to themselves. Many individuals keep in the straight path so long as they are supported in it. This is also the history of collective unities, and even of nations which, abandoned without a guide, present the spectacle of the most complete incoherence, or raise themselves to the sublime when a powerful and enlightened authority directs them. M. Renaudin relates the case of a patient who evinced a constant regret for the period when his confinement in a cell was attended by a more rigorous coercion. It was at this period, he says, that his power was greatest, most unlimited. *We should all have been saved if I had remained there*, he says. In proportion as his dwelling has been improved, his power has declined, and he is become the more docile by being allowed a larger measure of liberty. This remark cannot be lost sight of when the question of cells in asylums is considered. When cells were abandoned, all our patients, like the one referred to, lost the power which stimulated their excitation, and we are thus supplied with the solution of the most important problem in the organization of an asylum.

With these passages we must conclude our analysis of M. Renaudin's present series of essays. We cannot better show the high opinion we have formed of their merit, and of the instruction they convey upon some of the most initial, and therefore most difficult and important questions in medico-psychology, than by expressing an earnest hope that the author will not long defer the fulfilment of his promise to publish a continuation of his researches.

